# Increased levels of anti-BSA antibodies in children with Down syndrome

**DOI:** 10.3389/fendo.2023.1056925

**Published:** 2023-02-03

**Authors:** Sian L. Grace, Georgina L. Mortimer, Aizhan Kozhakhmetova, Jamie Leveret, Richard Newton, Koit Reimand, Julian P. H. Shield, Raivo Uibo, Alistair J. K. Williams, Kathleen M. Gillespie

**Affiliations:** ^1^ Bristol Medical School, University of Bristol, Bristol, United Kingdom; ^2^ Department of Neurology, Royal Manchester Children’s Hospital, Manchester, United Kingdom; ^3^ Department of Immunology, Institute of Bio- and Translational Medicine, University of Tartu, Tartu, Estonia; ^4^ National Institute for Health and Care Research (NIHR) Bristol Biomedical Research Centre, Nutrition Theme, University of Bristol, Bristol, United Kingdom

**Keywords:** Down syndrome, type 1 diabetes, autoimmune, islet autoantibodies, bovine serum albumin

## Abstract

**Introduction:**

Autoimmune diabetes occurs more often in the first 2 years of life in children with Down syndrome (DS) compared with the general population. We previously observed increased frequencies of islet autoantibodies, including insulin autoantibodies (IAA), in children with DS. Assays for IAA using ^125^I-labelled insulin require competition to overcome cross reactivity with antibodies to the cow’s milk protein, bovine serum albumin (BSA). ^125^I-IAA assay results suggested that levels of antibodies to BSA may also be increased in children with DS. The aim of this study therefore was to determine whether the levels of anti-BSA antibodies differed in children with DS compared with controls.

**Methods:**

Samples were available from two populations with DS: one from the UK, (UK DS cohort n=106, 58 male, median age 12.5 years) and one from Estonia (Estonian DS cohort: n=121, 65 male, median age 9.75 years). A UK control population was provided by sex and age-matched healthy siblings of probands participating in the Bart’s Oxford (BOX) family study of type 1 diabetes. A competitive-displacement radiobinding assay (RBA) and a Dissociation Enhanced Lanthanide Fluoroimmunoassay (DELFIA) were developed to measure and confirm anti-BSA antibody levels. HLA class II genotype was analysed by PCR using sequence specific primers (PCR-SSP).

**Results:**

Overall, levels of anti-BSA antibodies were increased in those with DS compared with controls (p<0.0001) but this was not HLA associated.

**Conclusion:**

Increased levels of anti-BSA antibodies may reflect a defect in immune maturation or increased gut permeability in children with DS, increasing their risk of developing autoimmunity.

## 1 Introduction

Children with Down syndrome (DS) are at increased risk of developing autoimmune conditions. Thyroid autoimmunity occurs in approximately one in three children with DS and one in ten develop coeliac disease (1, 2). In previous studies, we showed that 6% of children with DS have two or more circulating islet autoantibodies, highly predictive of future type 1 diabetes (T1D) (3). In a study of 134 individuals with DS and a clinical diagnosis of T1D, we demonstrated that early onset, decreased genetic susceptibility, and multiple autoimmunity are characteristic of T1D in DS (4). Recent investigations identified DS as a cause of permanent autoimmune neonatal diabetes that is not associated with genetic susceptibility to T1D, confirming our findings of early onset and decreased human leucocyte antigen (HLA) associations (5). Significantly more children with DS are bottle-fed and have a shorter duration of breast feeding (6). Reduced duration of breast feeding and early exposure to dietary proteins have been implicated as risk factors for islet autoimmunity and progression to T1D (7–9). The mechanisms underlying the increased risk of autoimmunity, early onset of T1D, and how early life factors may influence this remain under explored in children with DS.

In the general population antibodies to bovine serum albumin (BSA), a 69 kDa dietary protein derived from cows, have been observed in children (10, 11). Decline in titre of antibodies to BSA with age has been related to tolerance induction in healthy individuals (12, 13). Karjalainen and colleagues reported that many individuals with new onset T1D had serum anti-BSA antibodies; most directed against a 17-amino acid BSA peptide, ABBOS (14). Following up on this observation, Atkinson and colleagues showed that peripheral blood mononuclear cell responses to BSA were positive in only 2 of 24 new onset cases suggesting that BSA is not an antigen with a role in T1D pathogenesis (15). Presence of anti-BSA antibodies was not restricted to T1D; they were present in individuals with thyroiditis, systemic lupus erythematosus and rheumatoid arthritis (15).

Insulin autoantibodies (IAA) are key markers in identifying children at risk of T1D and are often the first to appear (16). In radiobinding (RBA) for IAA, the main requirement for competitive displacement was to overcome non-specificity caused by antibodies to BSA (17, 18). In our analysis of IAA in children with DS, 37/104 (36%) required competitive displacement compared to 5-6% of age-matched controls. We postulated that the high requirement for competitive displacement of IAA in children with DS was caused by increased levels of antibodies to BSA. The aim of this study was to 1) test whether circulating levels of anti-BSA antibodies are increased in children and adults with DS compared with the common dietary antigen, ovalbumin and 2) analyse anti-BSA antibody associations with T1D and HLA.

## 2 Materials and methods

### 2.1 Cohorts

#### 2.1.1 Children with Down syndrome from the UK (UK DS)

Serum samples from 106 DS individuals were available (median age 12.5 years - range 2-26 years; 58 male), genetic samples were available from 105 of these individuals. These individuals have been described previously (3).

#### 2.1.2 Individuals with Down syndrome and type 1 diabetes (DSD) from the UK (UK DSD)

Sera and genetic samples from 21 individuals with DS and T1D were available (median age 35.6 years - range 13.6-45.4 years; 9 male; median sample time from diagnosis of T1D 11.2 years - range 2.8-39.6 years). These individuals have been described previously (4).

#### 2.1.3 UK control populations

Unaffected, autoantibody negative first-degree relatives (FDR) from the population-based Bart’s Oxford family (BOX) study of T1D patients diagnosed under the age of 21 years were age and sex-matched with the UK DS children, Control Group 1, and with the UK DSD population, Control Group 2 (19). Serum samples from 135 individuals in Control Group 1 were available (median age 11.8 years - range 6.1 – 25 years; 82 male), genetic samples were available from 116 (86%) of these individuals. Serum samples from 42 individuals in Control Group 2 were available (median age 35.6 years - range 13.6-45.4 years; 16 male), genetic samples were available from 21 (50%) of these individuals.

#### 2.1.4 UK Type 1 Diabetes (T1D) population

Serum samples from 21 individuals from the BOX study were available (median age 35.5 years - range 11.2-51.8 years; 10 male; median sample time from diagnosis of T1D 13.7 years - range 0.6-42.1 years), genetic samples were available from 14 (66%) of these individuals.

#### 2.1.5 Children with Down syndrome from Estonia (Estonian DS)

Serum samples from 121 Estonian children with DS were available (median age 9.7 years - range 0.02–45.8 years; 65 male) (20).

Controls from Estonia: Sera from 50 individuals without T1D from Estonia were age and sex matched with the Estonian DS population (mean age 11.4 years; range 1.5-41 years; 27 males/23 females).

### 2.2 Anti-BSA antibody radiobinding assay (RBA)

In duplicate 2µl serum was added to 96 deep-well microtitre plate (Beckman Instruments (UK) Ltd, High Wycombe, Bucks, UK). To each well, 15,000 counts per minute (cpm) of ^125^I-labelled BSA (900 TBq/mmol) was added in 25µL assay buffer (50 mmol/l Tris, 1% Tween-20, pH 8.0). The BSA was labelled with ^125^I by the chloramine T method and desalted on a PD10 column (GE Healthcare) (21). Bound immunocomplexes were precipitated by addition of Glycine-blocked Protein A Sepharose (GB-PAS) (GE Healthcare, Little Chalfont, Bucks UK). Bound ^125^I-labelled BSA was measured using a gamma counter (Perkin-Elmer LAS, Beaconsfield, Bucks, UK). Quality control sera with a range of affinities to anti-BSA antibodies were used. Results were indexed by subtracting the cpm of the negative control and dividing by the positive control minus the negative control. The threshold of positivity was defined as the 90th percentile of age, sex, and nationality matched controls.

### 2.3 Anti-BSA antibody Dissociation Enhanced Lanthanide Fluoroimmunoassay (DELFIA)

In duplicate 2µl of serum was added to 96-deep well microtitre plate. The BSA was labelled using an Eu-N1 ITC chelate, with an aromatic isothiocyanato group as the reactive arm. To each well, 28.8ng/ml of labelled BSA (approximately 95,000 Europium (Eu) counts) was added in 25µl assay buffer (50 mmol/l Tris 0.9% NaCl with <0.5% Tween-20). GB-PAS was used to precipitate bound immunocomplexes. Fluorescence was detected on a Victor plate reader after the addition of DELFIA Enhancement Solution (Perkin Elmer). Results were indexed by subtracting the fluorescence of the negative control and dividing by the positive control minus the negative control. The threshold of positivity was defined as the 90th percentile of age, sex, and nationality matched controls.

### 2.4 Anti-ovalbumin antibody detection

Antibodies to a second dietary protein Ovalbumin were measured in serum from 25 individuals from the UK DS cohort (median age 12.7 years – range 9.3-17.4; 17 male) and a control group of 37 FDRs from the BOX study (median age 10.7 years – range 6.1-14.7; 18 male) using the same method as for anti-BSA antibody detection. Except for ^125^I-labelled ovalbumin (880 GBq/mmol), no ovalbumin was present in any other reagents used. Antibody binding was expressed as cpms.

### 2.5 HLA genotyping

DNA samples for HLA genotyping were available for the UK cohorts only. Methods of DNA extraction and HLA class II genotyping using sequence specific primers have been published previously (22). The haplotypes HLA-DRB1*04-DQB1*0302 or HLA-DRB1*03-DQB1*0201 were considered risk haplotypes for T1D (23).

### 2.6 Data analysis

Frequencies of positivity to anti-BSA antibodies were compared using Chi squared analysis and Fisher’s exact test where appropriate. Mann-Whitney U testing was used to compare anti-ovalbumin antibody binding. Results with a p-value <0.05 were considered significant. Q-Q Plots were used to describe the distribution of results.

## 3 Results

### 3.1 Anti-BSA antibody detection by RBA

**Figure 1:**
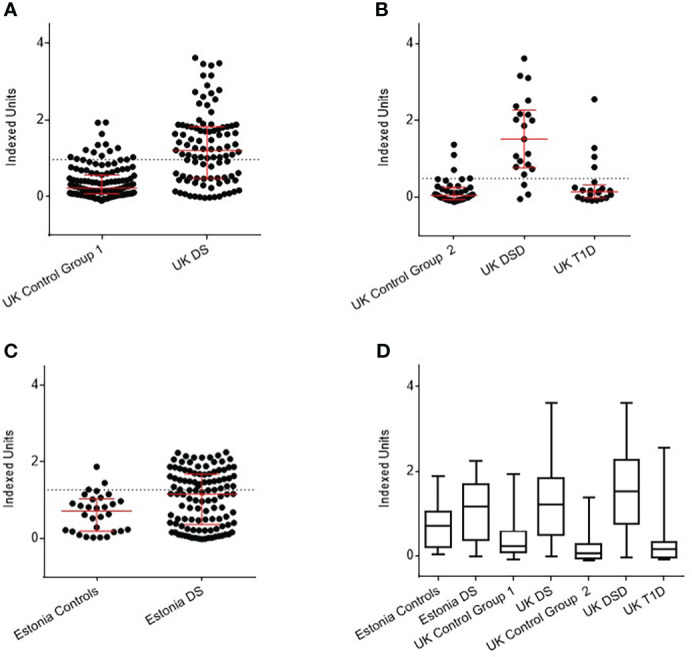
Detection of anti-BSA antibodies by radiobinding assay in Down syndrome (DS) populations compared with age, sex, and nationality matched controls. **(A)** UK DS have increased levels of anti-BSA antibodies compared to controls (p<0.0001). **(B)** UK DSD with T1D have increased levels of anti-BSA antibodies compared to controls (p<0.0001), those with T1D alone have similar levels of anti-BSA antibodies compared to controls. **(C)** Estonian DS have increased levels of anti-BSA antibodies compared to controls (p=0.0073). **(D)** Comparison of anti-BSA antibody titres detected by radiobinding assay between all cohorts. The dotted line represents 90^th^ centile of age, sex, and nationality matched controls. The red bar represents median and interquartile ranges. Whiskers indicate minimum to maximum. Indexed units were calculated by subtracting the cpm of the negative control and dividing by the cpm of the positive control minus the negative control. DSD, Patients with Down syndrome and T1D, Type 1 Diabetes.

The threshold for anti-BSA antibody positivity in UK DS children was 0.93 Indexed units (IU), as determined in 135 controls. Using this threshold, 52 of 106 UK DS children (49%) were found to be positive for anti-BSA antibodies, compared with 14 of 135 (10.4%) controls (p<0.0001) (**Figure 1A**). Of the 21 UK DSD individuals, using a positivity threshold determined by 42 controls of 0.48 IU, 17 (80.9%) were positive, compared with 4 (9.5%) controls and 5 of 21 (23.8%) T1D individuals (**Figure 1B**).

Of the 121 Estonian children with DS, using threshold for positivity as determined by 50 controls of 1.05 IU, 54 (44.6%) were positive for anti-BSA antibodies, compared with 6 (12%) controls (p<0.0001) (**Figure 1C**).

As an overview, anti-BSA antibody titres detected by RBA between all cohorts are shown in **Figure 1D**. The data are shown in tabular form in **Supplementary Table 1**.

### 3.2 Can increased levels of anti-BSA antibodies in DS be replicated using an independent method (DELFIA)?

**Figure 2:**
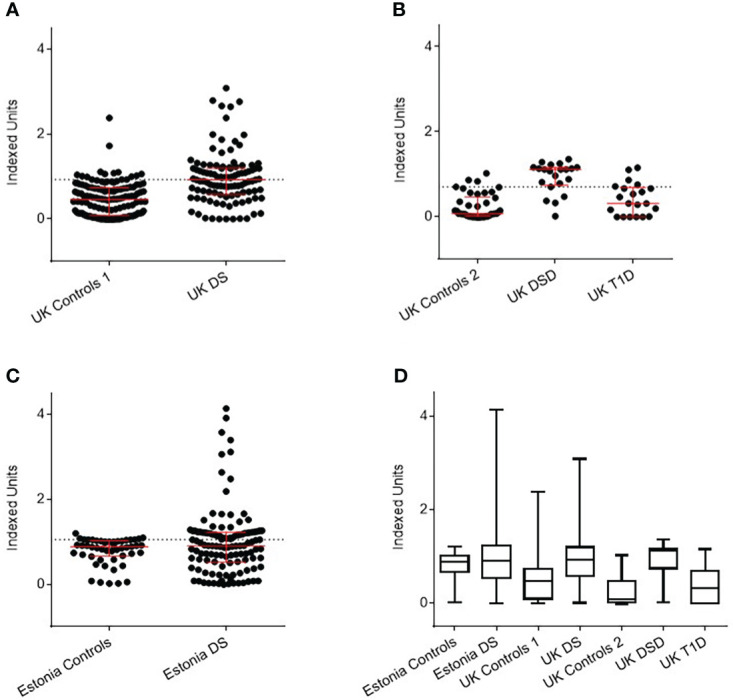
Detection of anti-BSA antibodies by DELFIA assay in Down Syndrome (DS) populations with age, sex, and nationality matched controls. **(A)** UK DS have increased levels of anti-BSA antibodies compared to controls (p<0.0001). **(B)** UK DSD with T1D have increased levels of anti-BSA antibodies compared to controls (p<0.0001), those with T1D alone have similar levels of anti-BSA antibodies compared to controls. **(C)** Estonian DS have similar median levels of anti-BSA antibodies compared to controls but 19 individuals with DS had elevated levels of anti-BSA antibodies. **(D)** Comparison of anti-BSA antibody titres detected by DELFIA assay between all cohorts. The dotted line represents the 90^th^ centile of age, sex, and nationality matched controls. The red bar represents median and interquartile ranges. Whiskers indicate minimum to maximum. Indexed units were calculated by subtracting the fluorescence of the negative control and dividing by the cpm of the positive control minus the negative control. DSD, Patients with Down syndrome and T1D, Type 1 Diabetes.

All UK samples were available for retesting with the DELFIA method. Of 106 UK DS children, using a positivity threshold determined by 135 controls of 0.97 IU, 59 (55.7%) were positive for anti-BSA antibodies, compared with 14 (10.4%) of controls (P<0.0001) (**Figure 2A**). Of 21 UK DSD individuals, using a positivity threshold determined by 42 controls of 0.49 IU, 18 (85.7%) were, compared to 6 (9.5%) controls and 4 of 21 (19%) individuals with T1D (p<0.0001) (**Figure 2B**). Not all Estonian samples had sufficient sample volume to undergo retesting with the DELFIA method. Of the 111 Estonian DS children, using a positivity threshold determined by 30 controls of 1.27 IU, 49 (44%) were positive, compared to 3 (10%) controls (p<0.05) (**Figure 2C**). The data are shown in tabular form in **Supplementary Table 1**.

Comparison of anti-BSA antibody titres detected by DELFIA between all cohorts are shown in **Figure 2D**.

### 3.3 Is the increased antibody response to BSA specific to all food antigens?

To determine whether the increased response to BSA in DS individuals reflected a generalised enhanced response to food antigens, a RBA was developed to measure anti-ovalbumin antibodies. No significant difference in the level of anti-ovalbumin binding was found between the 25 UK DS children (median 4828 cpm - range 1277-5982 cpm) and the 37 matched control samples (median 4596 cpm - range 1260-5461.6 cpm), data not shown. This analysis included testing individuals who had positive and negative responses to BSA in the previous experiments.

### 3.4 Is anti-BSA antibody positivity associated with HLA genotypes?

Of 106 UK individuals with DS, data on HLA and anti-BSA antibodies status was available on 105 but no difference was observed in T1D associated HLA risk genotypes and positivity for anti-BSA antibodies. Chi squared for trend p=0. 45 (**Table 1**).

**Table 1:** The frequency of high risk HLA haplotypes for type 1 diabetes were similar in individuals with DS with increased levels of anti-BSA antibodies.

	Anti-BSA antibody positive (n)	Anti-BSA antibody negative (n)
DRB1*04-DQB1*0302/ DRB1*03-DQB1*0201	2	2
DRB1*04-DQB1*0302/X	10	6
DRB1*03-DQB1*0201/X	11	10
X/X	29	35
Total	52	53

X denotes a non-risk haplotype.

## 4 Discussion

Increased levels of anti-BSA antibodies were identified in two independent cohorts of individuals with DS compared with matched controls initially using a RBA detection method. This was replicated when samples were re-analysed using a DELFIA detection method although there were some differences in levels of positivity between the assays. A DSD cohort was also tested using both methods and increased levels of anti-BSA antibodies were observed in the DSD cohort in comparison to healthy controls and those with T1D. In contrast, no immune response to ovalbumin was observed in individuals with DS, indicating that the responses observed are specific to BSA.

Two robust and sensitive assays to detect anti-BSA IgG antibodies were designed. Quality controls were standardized with reproducible index values. During assay development, the iodinated BSA label was found to have limited stability, with the quality often decreasing within a week. Therefore, a non-radioactive assay was developed with a more stable label and eliminating the disadvantages of using a RIA. This study has a reasonable sized sample pool in comparison to previous studies which have focused on the testing for anti-BSA antibodies. However, many of these previous studies have focused on anti-BSA antibody levels in T1D in comparison to control cohorts (15, 24). During a literature search, only one other study identified appears to have looked into anti-BSA antibodies in a DS cohort as well as a T1D cohort (25). In comparison to this study, our cohort sizes are much larger and are further validated by the inclusion of the DSD cohort and comparison with DS individuals of a different nationality, where the finding was replicated. In this study, FDR of individuals with T1D were chosen as controls. As such, similar to the DS population, they are also at increased risk of developing T1D. This suggests that the difference in anti-BSA antibody levels between DS and the general population is likely to be even greater than that observed in this study.

Immunoprecipitation of anti-BSA was achieved using GB-PAS which mainly binds IgG, and therefore our assay was not suited to measurement of IgM or IgA anti-BSA. IgA antibodies are traditionally associated with the gut and therefore titres of IgA antibodies could be affected by exposure to dietary proteins, such as BSA. However, in 10 samples tested for IgA anti-BSA, binding mirrored those of IgG anti-BSA (data not shown). There could be a possible influence of DS on mucosal Ig levels, which may not necessarily be represented by the altered serum IgG responses we detected. It has been found that whilst salivary Ig levels may differ between DS subgroups, there are no significant differences between salivary and serum Ig levels between patients with DS and healthy controls (26, 27).

Furthermore, antibodies to BSA are common in the UK and Estonian populations, with many of the individuals having increased levels of binding, and although we set our threshold for positivity at the 90th percentile to identify those with high levels of binding, additional work is needed to identify the optimum threshold that allows discrimination between a normal and an exaggerated humoral response to BSA.

There are well reported immune deficiencies in DS: the DS thymus is small with an abnormal structure, even in the neonate, and shows a decreased proportion of phenotypically mature thymocytes (28). Increased levels of anti-BSA IgG antibodies in individuals with DS may indicate an underlying immunological defect and this is supported by several observations: T cell receptor (TCR) excision circles (TREC) are episomal DNA circles generated during the process of V (D) J TCR gene rearrangement, and as they are not replicated during mitosis can be used as a measure of thymopoiesis. Reduced TREC counts in DS patients suggests they have a lower thymic output which may be exacerbated with age (29).

Furthermore, absolute numbers of T lymphocytes are also decreased in the DS neonate as well as the proliferative response to phytohaemaglutinin (PHA), implying a deficient reaction to antigenic stimulation (30, 31). In addition, reduced numbers of circulating B cells have been observed in patients with DS (32), despite comparable levels of circulating immunoglobulins. After stimulation with the TLR9 ligand CpG, cultured B cells showed increased differentiation into IgM plasma cells. Another study found that while normal germinal centres and transitional B cells were observed in individuals with DS, the number of CD27+ memory B cells was reduced and both natural effector and IgA memory B cells showed defects in maturation (33). Adaptive B-cell responses to pneumococcal polysaccharide vaccination were also impaired in DS patients (32, 34).

The autoimmune regulatory (AIRE) gene is a transcription factor on chromosome 21 which is essential for the expression of tissue specific antigens (TSA) for presentation to thymocytes (immature T cells) in the thymus and mature T cells in the periphery (35). These self-antigens are then presented to T cells by MHC complexes during the process of negative selection. T cells specific for self-antigens are deleted in this process. Without AIRE, self-reactive T cells to TSAs, are increasingly released into the periphery (36). It might be assumed that its expression would be increased in individuals with DS. The converse appears to be the case; analysis of protein and gene expression in surgically removed thymi from 14 patients with DS showed reduced expression of AIRE compared with 42 age-matched controls (37) but studies in DS have shown that the expression pattern in tissues is complex (38). Recent studies have highlighted the importance of hyper activation of interferon responses creating chronic inflammation in DS [Reviewed in (39)].

Early feeding regimes may influence the levels of antibodies to BSA in individuals with DS. Pisacane et al. reported that 57% of DS babies were bottle fed compared to 15-24% of healthy infants (6). Therefore, there is variation between the diets of DS children and those from the general population and indeed there may be differences between the UK and Estonian populations. Our study was not designed to examine the natural history of antibody development in children with DS. A longitudinal birth cohort study is required to determine the age at which antibodies to BSA appear in DS children. This should be accompanied by details on early feeding and clinical tests to measure gut permeability which may influence the levels of anti-BSA antibodies detected.

In conclusion, our previous studies had demonstrated increased risk of autoimmune diabetes in children with DS. This study identified a significant increase in positivity and titre of anti-BSA antibodies in children with DS compared with controls in two different European cohorts. This increase was observed in DS populations with and without T1D. Increased levels of anti-BSA antibodies may reflect an immune defect in maturation or increased gut permeability in children with DS. This study contributes to the distinct immunological characteristics of DS and future studies will examine longitudinal anti-BSA responses in the context of developing autoimmunity early in life.

## Data availability statement

The raw data supporting the conclusions of this article will be made available by the authors, without undue reservation.

## Ethics statement

The studies involving human participants were reviewed and approved by MREC/02/6/26. Written informed consent to participate in this study was provided by the participant or their legal guardian/next of kin.

## Author contributions

AW (deceased) and KG contributed to the original idea and with SG, GM, AK, JL, RN, KR, JPHS, and RU they designed the study and performed data collection; SG, GM, AK, and JL performed data analysis and wrote the manuscript with oversight by KG; KG is responsible for the integrity of the work as a whole. All authors contributed to the article and approved the submitted version.
