# Supplementary material for: Increased levels of anti-BSA antibodies in children with Down syndrome
Source: Front Endocrinol (Lausanne). 2023 Feb 3;14:1056925. doi: 10.3389/fendo.2023.1056925 (PMC9935828; doi:10.3389/fendo.2023.1056925)
Supplement: Supplementary file 1 [file Table_1.pdf]

	**UK DS-FDR**	**UK DS**	**UK DSD-FDR**	**UK DSD**	**UK T1D**	**Estonia Controls**	**Estonia DS**
RBA							
anti-BSA Positive<br><br>n (%)	14 (10.4)	52 (49.0)	4 (9.5)	17 (80.9)	5 (23.8)	6 (12.0)	54 (44.6)
anti-BSA titre<br><br>(IU Median and IQR)	0.46 (0.10, 0.73)	0.92 (0.59, 1.19)	0.07 (0.01, 0.42)	1.11 (0.78, 1.16)	0.31 (0.00, 0.66)	0.88 (0.68, 1.02)	0.91 (0.53, 1.23)
Delfia							
anti-BSA Positive<br><br>n (%)	14 (10.4)	59 (55.7)	6 (9.5)	18 (85.7)	4 (19.0)	3 (10.0)	49 (44.0)
anti-BSA titre<br><br>(IU Median and IQR)	0.22 (0.08, 0.56)	1.21 (0.48, 1.82)	0.04 (-0.06, 0.24)	1.51 (0.79, 2.17)	0.14 (-0.02, 0.25)	0.71 (0.20, 1.01)	1.16 (0.38, 1.67)

(Supplemental) Table 1: anti-BSA prevalence and levels in cohorts. Note the ages of the cohort vary as outlined in the methods section.
